# Intramuscular Hemangioma of the Forearm with Flexion Contracture

**DOI:** 10.1155/2019/6024039

**Published:** 2019-05-02

**Authors:** Sunyarn Niempoog, Waroot Pholsawatchai

**Affiliations:** Department of Orthopaedic Surgery, Thammasat University, 99 Moo 18, Khlong Nueng, Khlong Luang, Pathum Thani 12120, Thailand

## Abstract

Intramuscular hemangiomas can be an infrequent but important cause of musculoskeletal pain. This report describes a 25-year-old male who presented with pain in the right forearm and contracture of the right hand for 4 years. Physical examination revealed severe tenderness of the midforearm with contracture of the flexor tendons in the index, middle, ring, and little fingers. Conventional radiography of the forearm revealed a soft tissue phlebolith. Magnetic resonance imaging showed a well-defined lobulated mass partially involving the FDP tendon. An intramuscular hemangioma within the deep anterior compartment of the forearm was suspected. Following surgical excision of the hemangioma, the patient's symptoms resolved completely. In conclusion, intramuscular hemangioma can be a rare cause of flexion contracture of the hands and should be considered as a cause of a flexion contracture that fails to respond to conservative treatment.

## 1. Introduction

Hemangiomas are abnormal proliferations of blood vessels, making up 7% of all benign soft tissue tumors [1, 2]. Intramuscular hemangiomas are rare and account for <0.8% of all hemangiomas [3]. Chronic pain and a new mass are the most common presenting symptoms [4–6], and a muscle contracture may be present. Rounded soft tissue calcifications may be seen on X-ray, termed phleboliths, but MRI is the diagnostic procedure of choice [7]. Multiple treatments are available for symptomatic intramuscular hemangiomas, and options include conservative management, systemic corticosteroids, embolization, radiation, sclerotherapy, and surgical excision [8, 9]. Nonoperative treatment may decrease symptoms as the malformation involutes, including activity modification, compression garments, elevation of the extremity, nonsteroidal anti-inflammatory drugs, and physical therapy [5]. Surgical intervention is indicated in rapid tumor growth, intractable pain, risk of local skin necrosis, thrombocytopenia, cosmetic or functional impairment, or suspicion of malignancy [2, 10, 11]. Incomplete surgical excision is the greatest risk factor for recurrence, and inadequate excision can result in debilitating complications [10, 12]. Sclerotherapy, corticosteroids, radiation therapy, or embolization may be beneficial in complex, high-risk infiltrating lesions [4, 6, 13].

## 2. Case Report

A 25-year-old man presented with a painful flexion contracture of his right hand for four years. 10 years earlier, he had a motorcycle accident, injuring the right forearm. Following the injury, he had pain in his right forearm for several days, which improved with nonsteroidal anti-inflammatory drugs (NSAID). He was asymptomatic and had no movement deficits after the medication. Over the next few years, he gradually developed stiffness in his right index, middle, ring, and little fingers with pain in his right forearm and increasing difficulty in using the affected hand. He was unable to extend his metacarpophalangeal (MCP) joints, proximal interphalangeal (PIP) joints, and the distal interphalangeal (DIP) joints of the index, middle, ring, and little fingers. There were no constitutional symptoms like fever or weight loss.

**Figure 1:**
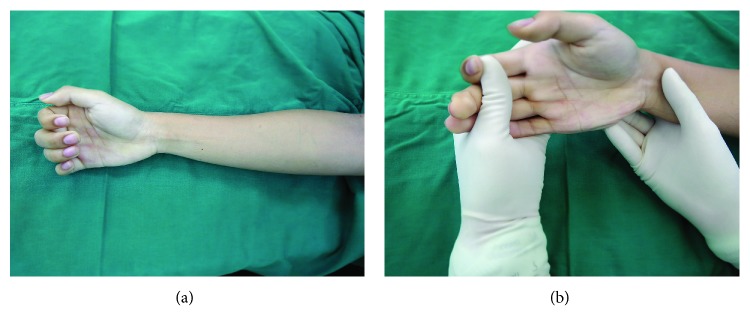
Preoperative physical examination revealed the flexion contracture of fingers in resting position (a). Passive extension on right index, middle, and ring fingers on the PIP joint and DIP joint was grossly limited (b).

On local examination, his right midforearm was tender, and no mass or swelling could be palpated. His right index, middle, ring, and little fingers were held in flexion, and passive extension of the PIP and DIP joints was grossly limited (Figure 1). The pain was worse on passive extension of the finger, especially his index finger. Active and passive flexion of the index, middle, ring, and little fingers was unaffected. There was no motor weakness and no sensory deficit.

**Figure 2:**
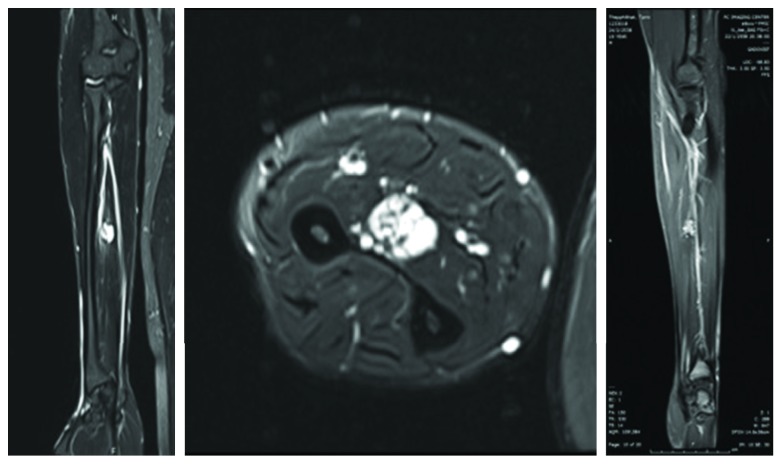
MRI showed the hemangioma.

A clinical diagnosis of myositis flexion contracture was made. Conventional radiography revealed a soft tissue mass with calcific spots. Magnetic resonance imaging showed a well-defined lobulated mass with a bright signal intensity (SI) on T2W. Internal septations and dark internal SI spots, consistent with calcifications and/or hemorrhage, were present midway on the lateral aspect of the flexor digitorum profundus (FDP) tendon and partially involved the FDP muscle and extended to the intermuscular plane between the FDP and flexor pollicis longus (FPL). It was fed by an artery from the anterior interosseous artery, but it was not dilated (Figure 2).

**Figure 3:**
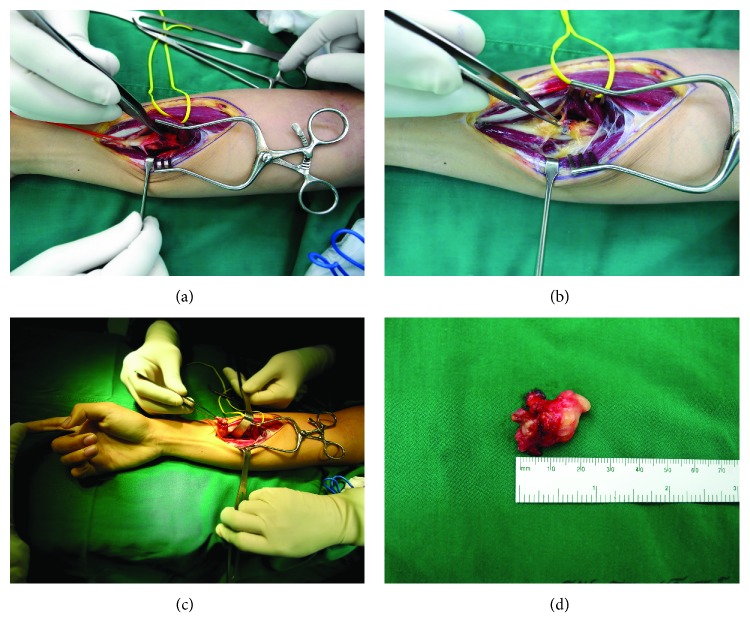
Surgery revealed the intramuscular mass infiltrating the FDP of the right forearm (a, b). The passive extension of the index finger was established (c). The heterogeneous, lobulated, rubbery, red-yellow mass (2 × 2 cm) consisted of hemorrhage, vascular invasion, and central necrosis (d).

**Figure 4:**
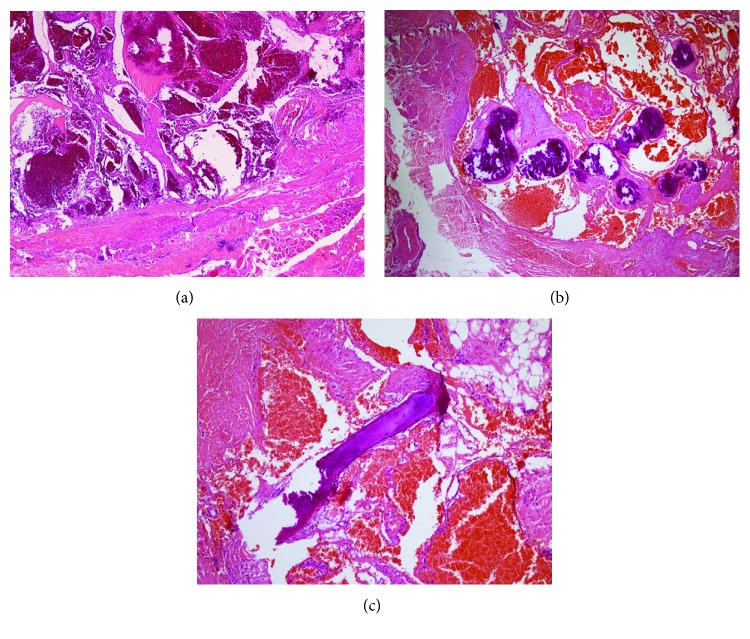
Microphotograph of intramuscular hemangioma. The lesion is an ill-defined intramuscular mass composed of proliferation of predominant cavernous-type vascular channels (40x) (a).The vascular channels are lined by variably thick walls and contain blood components. Foci of phleboliths are identified (100x) (b). Metaplastic ossification is noted (400x) (c).

**Figure 5:**
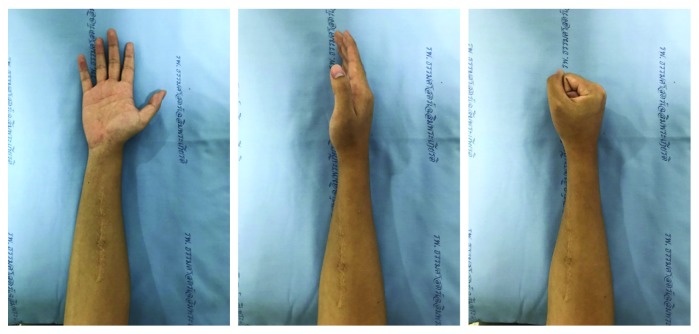
Active flexion and extension of MCP, PIP, and DIP joints of index, middle, ring, and little fingers, one year after surgical excision.

The patient underwent an excision of the mass. Surgery revealed a multiloculated red-yellowish mass, measuring 2 × 2 cm, which involved the FDP, but there was no neuromuscular involvement (Figure 3). After excision, passive extension of the index, middle, ring, and little fingers was established before skin closure (Figure 4). The final pathologic diagnosis was intramuscular hemangioma with the presence of thrombosis and a phlebolith (Figure 5). Postoperatively, the patient could actively extend his right index, middle, ring, and little fingers. Full functional restoration was achieved approximately 1 month after surgery.

## 3. Discussion

Hemangiomas are one of the most common benign soft tissue tumors [14, 15]. Less than 1% of all hemangiomas are deep-seated hemangiomas such as intramuscular hemangiomas [15, 16]. Most intramuscular hemangiomas are usually present with a pain, swelling, palpable mass, and impaired extremity function [17–19], but to our knowledge, contracture of infiltrated muscles is rare.

Plain radiography, ultrasound, and MRI may be helpful in the evaluation of intramuscular hemangiomas. The plain radiography may show the abnormal soft tissue shadows or associated bony erosion when lesions increased in size [20]. The rounded soft tissue calcifications, in terms of phleboliths, may be seen only in 25% of cases [11]. The hyperechogenic region or acoustic shadowing of calcified phlebolith in ultrasound may be useful to identify the hemangiomas [15]. The MRI is the modality of choice to diagnose hemangiomas. The lesions have hyperintense signal compared to skeletal muscle on T2-weighted images, relating to dilated vascular spaces filled with stagnant blood. On T1-weighted images, 70% of lesions have only mildly hyperintense signal relative to skeletal muscle. Diagnosis is usually made following a clinicoradiological examination. The biopsy is rarely needed.

Multiple treatment options are available for symptomatic intramuscular hemangiomas. Some of the intramuscular hemangiomas may involute with time but do not undergo spontaneous regression [15, 21]. Surgical intervention is indicated in rapid tumor growth, intractable pain, risk of local skin necrosis, thrombocytopenia, cosmetic or functional impairment, or suspicion of malignancy [2, 10, 11, 15].

Few of the literatures have reported intramuscular hemangioma with contracture in the upper extremity. Kamath et al. [22] reported intramuscular hemangioma involving only the flexor digitorum superficialis (FDS) of the ring finger, managed with total excision and end to side anastomosis of the FDS to the middle finger. Sunil [23] reported an intramuscular hemangioma in a 12-year-old girl, who presented with a painful mass in the left forearm and Volkmann-like contracture, which restricted the finger movements in active flexion and extension of all fingers. Full functional restoration was achieved after surgical excision. The case we are describing is unique in the fact that there was no palpable mass, and the contracture of all digits presented only in finger extension, but the flexion was unaffected.

In our case, it took four years to establish the correct diagnosis of the intramuscular hemangioma. Physical examination revealed pain with stiffness of the index, middle, ring, and little fingers but no palpable mass. Such vague symptoms and signs can lead to a misdiagnosis of trigger finger and inappropriate treatments by many health care providers.

However, upon correct diagnosis by MRI and successful excision, the patient's symptoms and signs resolved fully, and he had normal hand function.

In conclusion, this case demonstrates that when a patient presents with flexion contracture of the forearm with or without pain and fails to respond to conservative treatment, intramuscular hemangioma or other soft tissue tumors should be included in the differential diagnosis. Appropriate imaging is essential to establish the diagnosis.
